# Rectus Sheath Blocks for Umbilical Hernia Reductions in the Emergency Department: A Case Series

**DOI:** 10.5811/cpcem.7233

**Published:** 2024-07-31

**Authors:** Katherine Vlasica, Amanda Hall

**Affiliations:** St. Joseph’s Health, Paterson, New Jersey

**Keywords:** *rectus sheath block*, *umbilical hernia*

## Abstract

**Introduction:**

Rectus sheath blocks have been used for decades in the operating room for analgesia following umbilical surgical procedures. We present the first reported case series of a rectus sheath block used in the emergency department (ED) for the reduction of an umbilical hernia.

**Case Series:**

Four patients presented to the ED for painful, non-reducible umbilical hernias. An ultrasound-guided bilateral rectus sheath block was used in all four patients with complete pain relief and an easy hernia reduction.

**Conclusion:**

Rectus sheath blocks are an excellent addition to a multimodal analgesic regimen in periumbilical pain and painful procedures. This block is easy to perform and implement for pain control in umbilical hernias in an ED setting.

Population Health Research Capsule What do we already know about this clinical entity?

*Bilateral rectus sheath blocks have been used for decades in the operating room for periumbilical surgical anesthesia.*

What makes this presentation of disease reportable?

*This is the first reported case series of a bilateral rectus sheath block used in the ED for bedside reduction of an incarcerated umbilical hernia.*

What is the major learning point?

*The bilateral rectus sheath block can be safely performed in the ED and should be added to the procedural armamentarium of emergency physicians.*

How might this improve emergency medicine practice?

*Bilateral rectus sheath block performed in the ED in lieu of procedural sedation provides targeted analgesia in a patient with an incarcerated umbilical hernia.*

## INTRODUCTION

Umbilical hernias are a common entity presenting to the emergency department (ED) and frequently present a pain management challenge for the emergency physician. Umbilical hernias account for 6–14% of all adult abdominal wall hernias and are more common in women and individuals with increased intraabdominal pressure as in pregnancy, obesity, or ascites.^1^ Patients with increased abdominal pressure typically have underlying pathology, making any surgical intervention a high risk for complications. Of note, 20% of all cirrhotic patients will develop an umbilical hernia, which makes a surgical intervention or procedural sedation deemed high risk for complications.^1^ This case series examines how we can use a bilateral rectus sheath block (BRSB) for safe and efficient ED umbilical hernia reduction.

**Image 1.**
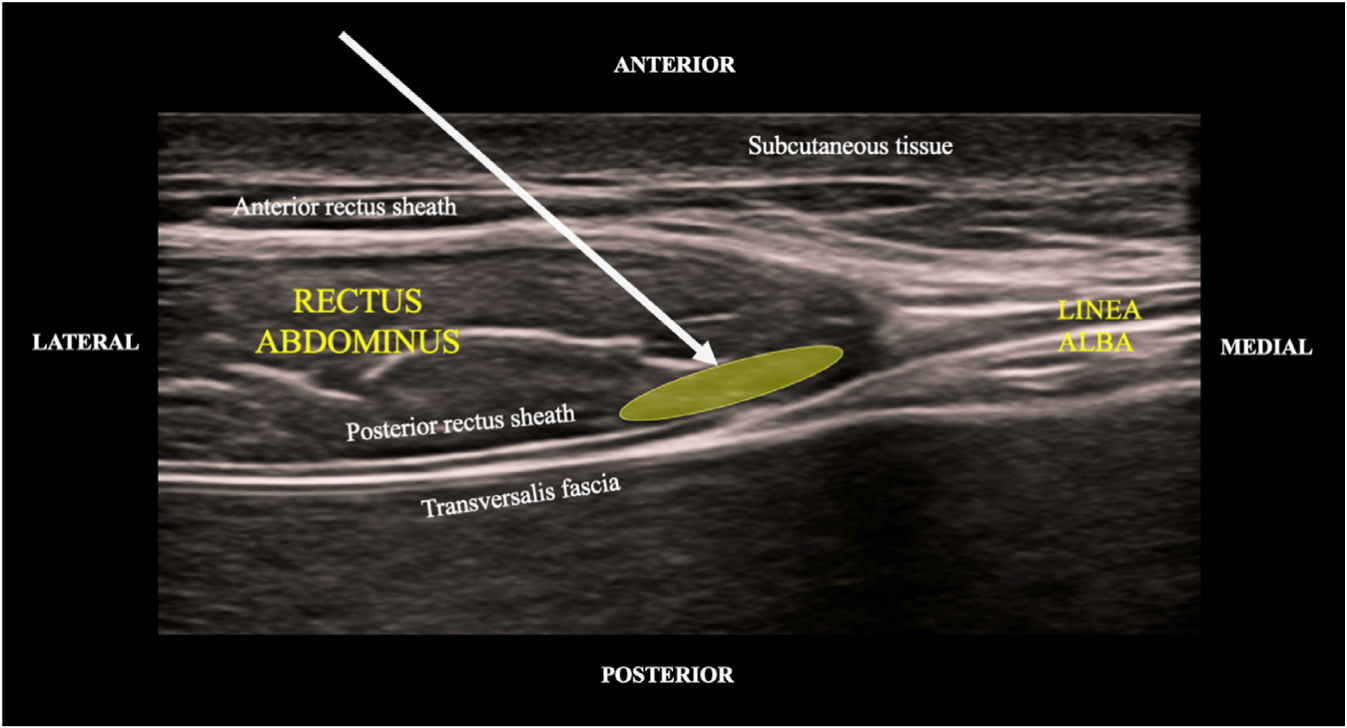
Point-of-care ultrasound demonstrating anatomical landmarks in the rectus sheath block. Yellow oval denotes the target area of injection. Arrow represents relative angle of trajectory for the needle.

The BRSB was first described in the literature in 1899. Throughout its lifespan the BRSB was primarily used in the operating room (OR) as a targeted analgesic intervention reducing postoperative nausea, vomiting, constipation, and opioid consumption.^2^ ^,^ ^3^ ^,^ ^4^ A BRSB provides midline analgesia to the rectus muscle and overlying skin from the xiphoid process to the symphysis pubis. The rectus abdominis muscle is a medial oval-shaped muscle that lies inside its rectus sheath formed by the split aponeurosis of the external oblique, transversus abdominus, and internal oblique, bordered by the linea alba medially and the linea semilunaris laterally. The ninth through eleventh intercoastal nerves, epigastric artery and veins are in the space between the rectus abdominis muscle and its posterior rectus sheath. Deep to the rectus sheath is the transversalis fascia and abdominal cavity containing peritoneum and abdominal viscera. In the rectus sheath block 0.1 milliliters (mL) per kilogram (kg) to a maximum of 10 mL per side is deposited bilaterally, resulting in a total volume of 20 mL of local anesthetic. The local anesthetic is deposited between the posterior rectus sheath and rectus abdominus muscle to allow for a successful block (Image 1). The block typically works unilaterally from approximately the seventh through eleventh thoracic level.^4^

## CASE SERIES

All four patients underwent the same technique for a BRSB. Consent was obtained prior to the procedure, and the patient was placed on a cardiac monitor. With the skin disinfected, a transducer was placed in a transverse axis above the umbilicus slightly lateral to the midline. Color Doppler was used to identify and avoid the epigastric arteries. A blunt-tipped hyperechoic block needle was inserted in plane through the subcutaneous tissue, anterior rectus sheath, and rectus muscle until resistance was felt as the blunt-tipped needle contacted the posterior rectus sheath (Image 1). Initially, 1–2 mL of saline were used to confirm placement after a heme-negative aspiration. We then instilled 10 mL of 0.5% ropivacaine under the posterior aspect of the rectus muscle, effectively lifting it from the posterior rectus sheath (Video). The process was repeated on the contralateral side. Each patient received a total of 20 mL of 0.5% ropivacaine, totaling 100 milligrams (mg) of ropivacaine. Maximum dose of ropivacaine is 3 mg/kg. All the patients were well within the safety margin of maximum dosing.

The video is a SonoClip (SonoClipShare.com) demonstrating the relevant anatomy and rectus sheath block performance. An echogenic block needle enters the rectus sheath from a lateral to medial direction. The target area of injection is between the underside of the rectus abdominus and posterior rectus sheath. An anechoic local anesthetic is seen lifting the rectus abdominus muscle from the posterior rectus sheath.

### Case One

A 60-year-old male with a past medical history of chronic umbilical hernia and alcohol use disorder presented to the ED following a fall resulting in a non-reducible umbilical hernia and left ankle pain. The patient was given 400 mg of ibuprofen orally and 15 mg of ketorolac intravenously (IV) for his ankle pain caused by a distal fibular fracture. His ankle pain was controlled, but he still had pain with bedside reduction attempts. After the BRSB the umbilical hernia was easily reduced. The patient was discharged home for medical optimization and an elective umbilical hernia repair at the patient’s convenience.

### Case Two

**Image 2.**
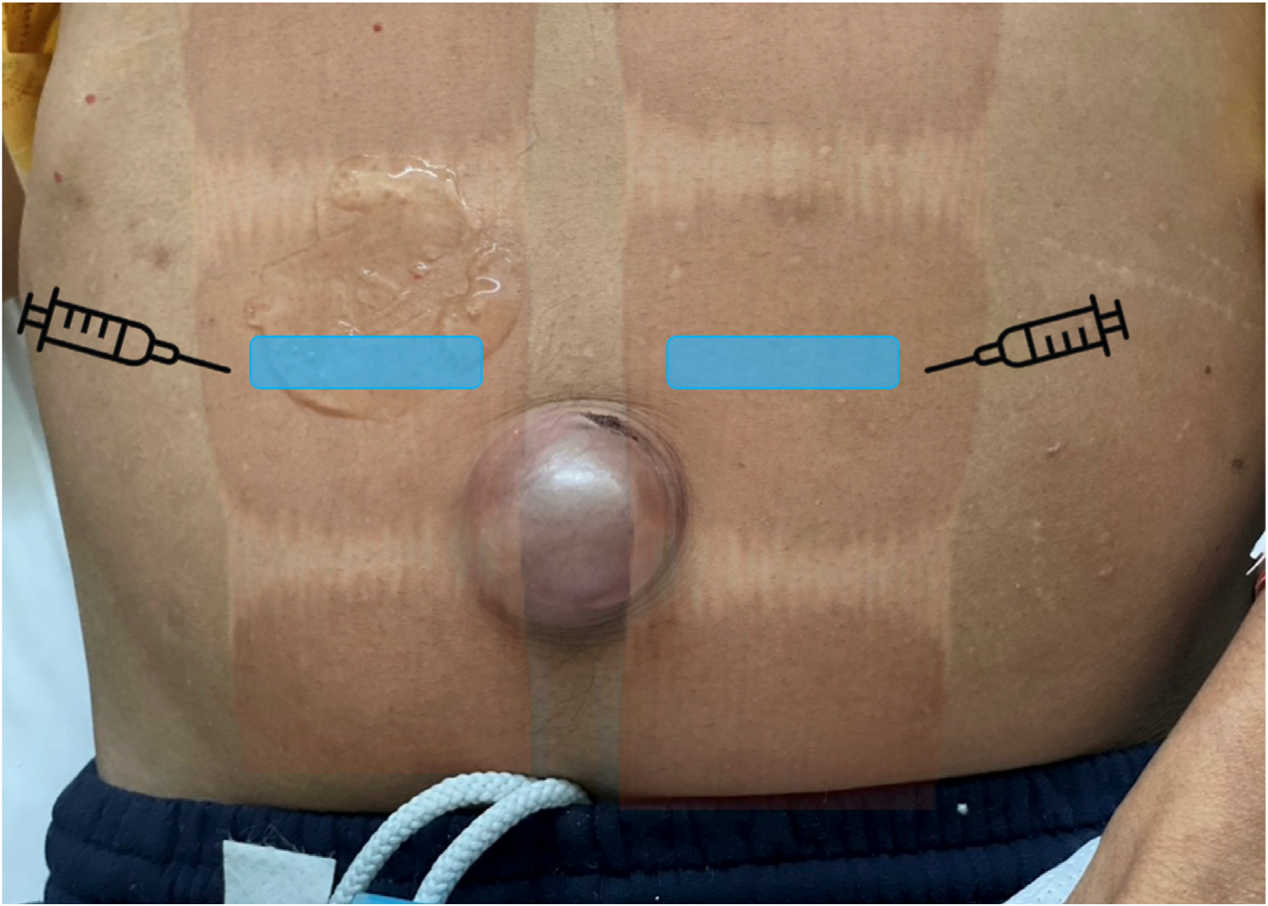
Patient with an incarcerated umbilical hernia prior to the bilateral rectus sheath block performed for pain control. Blue boxes indicate footprints of linear probe for a bilateral rectus sheath block. The needle is inserted in the lateral to medial direction, in plane with the linear transducer probe.

A 60-year-old male with past medical history of opioid abuse, ascites secondary to alcoholic cirrhosis, inguinal hernia, and umbilical hernia presented to the ED with complaints of gradually worsening severe abdominal pain with a worsening umbilical bulge (Image 2). The patient was initially given fentanyl 100 micrograms IV and haloperidol 5 mg IV for pain and nausea. There was an obvious umbilical hernia identified with skin changes and abdominal tenderness. Computed tomography (CT) of the abdomen and pelvis showed an umbilical hernia containing a small-bowel loop resulting in a small-bowel obstruction. Incarceration was unable to be excluded on CT. Surgery was consulted, and the patient was scheduled to undergo an emergent operative repair. Due to continued severe pain a BRSB was performed. Shortly after the block the patient reported significant improvement of pain and was transferred to the preoperative area. Three hours later the patient was re-evaluated in the preoperative area by the surgical team; the patient’s hernia had spontaneously reduced, and he was pain free and resting comfortably. Given the patient’s numerous comorbidities, the decision was made to postpone the surgery for medical optimization. The patient was monitored in the hospital for a few days, normal bowel patterns returned, pain did not resume, and he was discharged home four days after initial presentation. After the BRSB the patient did not require any additional analgesia.

### Case Three

A 49-year-old female with a past medical history of obesity, hypercholesterolemia, non-insulin-dependent diabetes, and hypertension presented to the ED for abdominal pain at the site of a previous umbilical hernia repair. The patient stated she had been admitted multiple times for pain control and discharged with instructions to schedule an elective operative repair as outpatient. Physical exam showed an obese female with mild tenderness to palpation of the umbilicus. The patient took acetaminophen 975 mg at home prior to arrival and received ketorolac 15 mg IV in the ED with improvement of pain but had persistent tenderness to palpation of hernia. A BRSB was performed in the ED. The patient’s hernia spontaneously reduced, and she had complete resolution of her pain. She was discharged for outpatient follow-up with her general surgeon.

### Case Four

The last case was a 42-year-old male with no past medical history who presented to the ED for eight months of intermittent umbilical pain,that had worsened over the prior three days. He had not sought medical care prior to arrival in the ED. On exam he had a tender umbilical, non-reducible hernia, approximately 4 centimeters (cm) in size. Computerized tomography revealed a 3 × 3.7 × 3.4 cm, fat-containing umbilical hernia with a narrow neck measuring 15 millimeters. Initial bedside reduction was unsuccessful after administration of ketorolac 15 mg IV, placement in Trendelenburg position, and application of ice to the umbilicus. Surgical consult was called, and after discussion with the surgical team the patient underwent a BRSB. Approximately 20 minutes after the block the hernia was reduced at bedside without difficulty, and the patient was discharged home with outpatient surgical follow-up.

## DISCUSSION

The BRSB has been used in the OR for decades to provide sensory analgesia to the anterior abdominal wall. In the past the rectus sheath block was performed without visualization, using a loss-of-resistance approach as the blunt-tipped block needle passed through fascial and muscle planes of the rectus sheath to anesthetize the anterior divisions of the seventh thoracic to first lumbar spinal nerves. This procedure was typically not performed in the ED due to fear of epigastric vessel puncture or bowel perforation due to inaccurate needle placement. However, the rise of ultrasound use in ED procedures over the last decade has led to the adaptation of many ultrasound-guided regional anesthesia (UGRA) techniques from their OR provenance. The ability to visualize the distribution of analgesic medications around and near nerves has increased accuracy of these blocks and limited accidental injection into vasculature or damage to surrounding anatomy. To the best of our knowledge, these four cases represent the first reported use of BRSB for the management of an umbilical hernia in the ED.

Given the rising rates of substance use disorders in the US, the spotlight has been placed on opioid usage and prescribing. Physicians are constantly evaluating novel methods of targeted analgesia for painful ED conditions as a part of a multimodal pain regimen. As demonstrated in this case series, we believe BRSB is a valuable tool for control of umbilical hernia pain in the ED. All four patients had complete resolution of their pain with minimal use of opioids, allowing for an either spontaneous or manual reduction of umbilical hernias with significantly less procedural discomfort to the patient.

The rectus sheath block can be performed quickly and safely at the bedside, providing rapid pain relief without parenteral opioids or the large mobilization of resources required for procedural sedation. All the procedures were performed by an emergency medicine resident and an UGRA-credentialed emergency attending physician, proving the safety, ease of performance and generalizability of this case series.

Ultrasound-guided regional anesthesia has proven to have numerous advantages over parenteral analgesic therapies for patients presenting with painful injuries to the ED: decreased incidence of central sensitization leading to chronic pain^5^; decreased adverse effects such as hypoxia, nausea and vomiting^6^ ^,^ ^7^; reduced opioid requirements^8^ ^,^ ^9^; and decreased length of stay.^10^ ^,^ ^11^ In multiple, published anesthesia literature cases it has been demonstrated that BRSBs were performed as the primary anesthetic in high-risk patients in cases of both inguinal and umbilical hernia repair with success.^4^ In this ED case series, a BRSB provided an expeditious and safe resolution of the patient’s pain with no complications, minimal opioid utilization, and no need for a full procedural sedation. While there are risks to both procedural sedation and UGRA, the risks of procedural sedation and parenteral opioid medications are more complex and potentially severe in the patient population typically prone to umbilical hernias. All patients avoided emergent operative repair subsequent to successful reduction of their umbilical hernias, enabling them to be discharged with outpatient scheduling for medical optimization.

## CONCLUSION

Ultrasound-guided regional anesthesia allows physicians to treat numerous acutely painful conditions and painful procedures in a timely, expeditious, and safe manner. The above cases describe the use of UGRA for the reduction of umbilical hernias without the need for procedural sedation or emergent surgical intervention. In all four cases the pain was controlled with minimal ED use of opioids, and the hernia was reduced without procedural sedation. Without this intervention the patients may have required emergent operative repair rather than an elective outpatient repair.

As emergency physicians expand their knowledge base and skillset, UGRA including the rectus sheath block is a technique we can use with hernia reduction, abdominal wall abscess, and any other painful periumbilical conditions. These four cases demonstrated the utility of a bilateral rectus sheath block in averting the need for procedural sedation and emergent operative repairs from the ED, also resulting in decreased resource utilization, opioid consumption, and length of stay. Overall, the BRSB is a great addition to the armamentarium of the emergency physician as an expeditious pain control option for periumbilical conditions.

## Supplementary Information

Video.
